# Data of the interacting protein networks and nucleotide metabolism pathways related to NDK and NT5

**DOI:** 10.1016/j.dib.2016.11.029

**Published:** 2016-11-17

**Authors:** Dan Zhang, Wen Ma, Yu He, Gu He, Peng Zhang, Hongxia Zhu, Ningzhi Xu, Shufang Liang

**Affiliations:** a State Key Laboratory of Biotherapy and Cancer Center, West China Hospital, Sichuan University/National Collaborative Innovation Center for Biotherapy, Chengdu, 610041, PR China; b Department of Urinary Surgery, West China Hospital, West China Medical School, Sichuan University, Chengdu, 610041, PR China; c Laboratory of Cell and Molecular Biology & State Key Laboratory of Molecular Oncology, Cancer Institute & Cancer Hospital, Chinese Academy of Medical Sciences, Beijing, 100034, PR China

**Keywords:** NDK, NT5, Network, Purine metabolism

## Abstract

The data presented in this article are related to the research article entitled “Antibacterial mechanism of daptomycin antibiotic against *Staphylococcus aureus* based on a quantitative bacterial proteome analysis” (Ma et al., 2016) [1]. Nucleoside diphosphate kinase (NDK) and 5′-nucleotidase (NT5) are two proteins related to bacterial growth. Here, a bioinformatics analysis was presented to explore NDK and NT5-invovled in the interacting protein network and purine metabolism.

**Specifications Table**

**Table:** 

Subject area	*Biology*
More specific subject area	*Microbiology*
Type of data	*Images, Text files*
How data was acquired	*Bioinformatics analysis obtained by STRING, and KEGG tools*
Data format	*Analyzed*
Experimental factors	*Software*
Experimental features	*Protein-protein interactions*, purine metabolism.
Data source location	*State Key Laboratory of Biotherapy, Sichuan University, Chengdu, China*
Data accessibility	*Data are available within this article*

**Value of the data**

• The data provide the protein interaction map related to NDK and NT5.
• The NDK and NT5-mediated metabolism network helps to understand their molecular functions in purine synthesis and degradation process.

## 1 Data

**Fig. 1:**
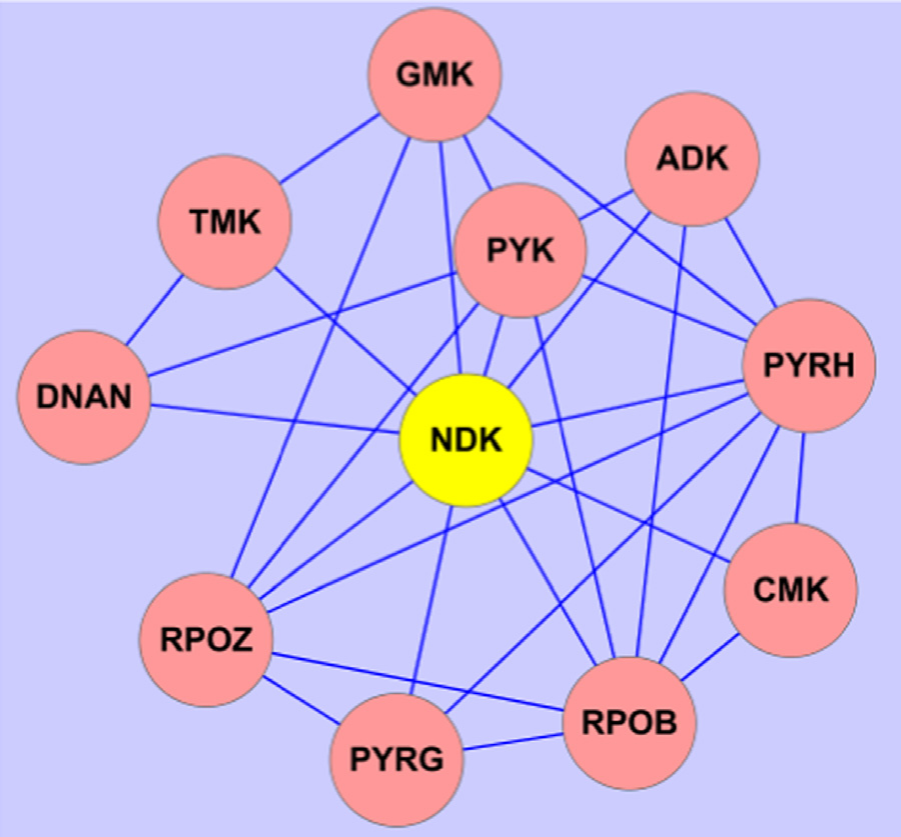
The NDK-interacting proteins. NDK, presented in yellow node, interacts with other proteins in pink nodes. NDK, nucleoside diphosphate kinase; TMK, thymidylate kinase; PYRG, CTP synthetase; RPOB, DNA-directed RNA polymerase subunit beta; PYRH, uridylate kinase; CMK, cytidylate kinase; DNAN, DNA polymerase III subunit beta; RPOZ, DNA-directed RNA polymerase subunit omega; GMK, guanylate kinase; PYK, pyruvate kinase.

**Fig. 2:**
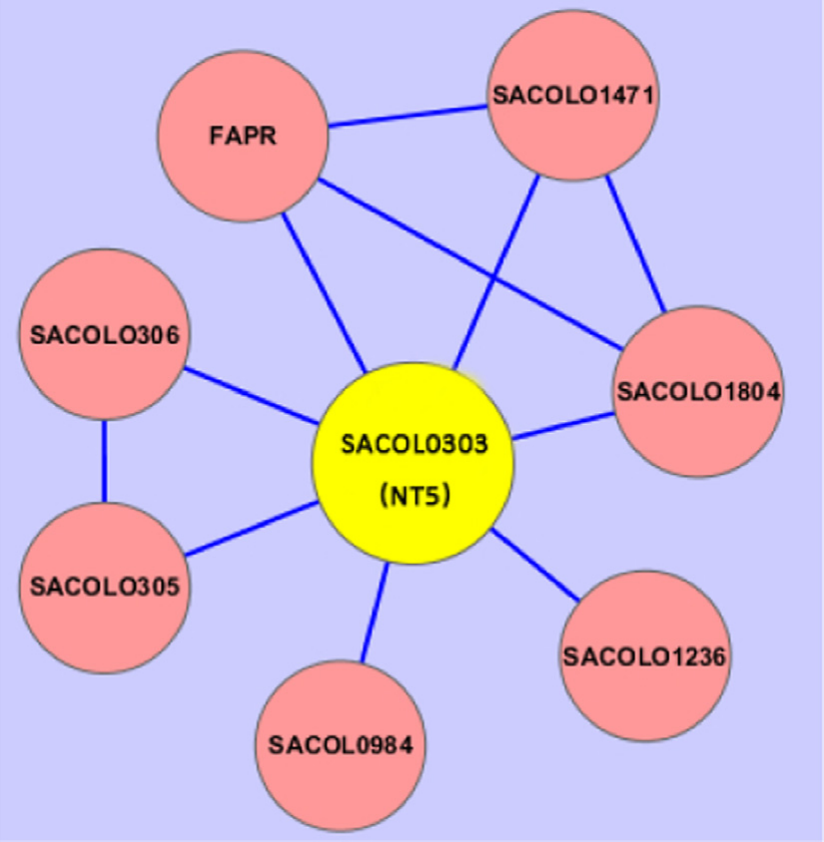
The NT5-interacting proteins. NT5, presented in yellow node, interacts with other proteins in pink nodes. NT5, 5’-nucleotidase; SACOL1804, polysaccharide biosynthesis protein; SACOL1471, putative cell wall enzyme EbsB; FAPR, fatty acid biosynthesis transcriptional regulator; SACOL1236, a 213 amino acid (aa) hypothetical protein; SACLO0984, a 171 aa hypothetical protein; SACOL0305, ABC transporter; SACLO0306, ATP-binding protein.

**Fig. 3:**
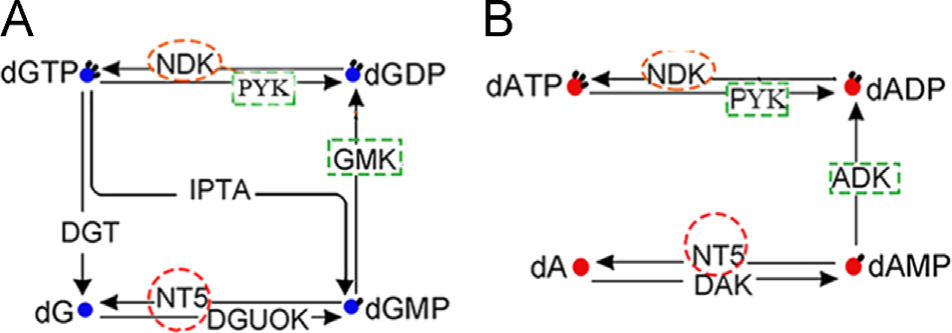
NDK and NT5 involve in purine metabolism pathways. NDK, NT5 and their interacting proteins involve in purine metabolism. PYK, pyruvate kinase; GMK, guanylate kinase; DGT, deoxyguanosine triphosphatase; IPTA, inosine triphosphate pyrophosphatase. DGUOK, deoxyguanosine kinase; dG,deoxyguanosine; dGMP, deoxy guanosine monophosphate; dGDP, deoxy guanosine diphosphate; dGTP, deoxy guanosine triphosphate; dA, deoxyadenosine; dAMP, deoxy adenosine monophosphate; dADP, deoxy adenosine diphosphate; dATP, deoxy adenosine triphosphate.

The interacting partners with NDK mainly belong to a serial of kinases (Fig. 1). The NT5 is associated with several biosynthesis and hypothetical proteins (Fig. 2). The two proteins locate in Map00230 which is named as purine metabolism (Fig. 3), one type of nucleotide metabolism in KEGG pathway database. Meanwhile, several of the proteins presented in interacting networks, including NDK, NT5, PYK, GMK and ADK, take part in the purine metabolism (Fig. 3). Data were obtained by bioinformatics analysis.

## 2 Experimental design, materials and methods

### 2.1 Protein-protein interaction prediction

The two proteins were imported into the online STRING (http://string-db.org/cgi/input.pl) separately, and searched by protein name SCAOL0303 (NT5) and NDK. The proteins interacting with NDK or NT5 were auto-analyzed by STRING tool.

The software Cytoscape was used to present the networks. According to the networks showed in STRING, NDK and NT5 were illustrated as graphs. In the graphs, the yellow nodes represent NDK or NT5, the pink nodes represent the interacting proteins with the two proteins, and the lines stand for the interaction relationship. The arrangement of nodes is applied to the ‘‘Spring Embedded’’ layout in Cytoscape.

### 2.2 Analysis of purine metabolism related to NDK and NT5

The NDK and NT5-mediated biological pathways were analyzed based on the KEGG database online (http://www.kegg.jp/kegg/pathway.html). NDK and NT5, two bacterial proteins sensitive to antibiotic daptomycin treatment [1], are marked with red dotted lines in metabolism pathway. Proteins interacting with NDK or NT5 are shown in green dotted line. Arrows in the pathway indicate molecular relationship. The blue spot and red spot represents deoxyguanosine and deoxyadenosine respectively, and black spot represents phosphoric acid.
