# Supplementary material for: Data of the interacting protein networks and nucleotide metabolism pathways related to NDK and NT5
Source: Data Brief. 2016 Nov 17;9:1063–6. doi: 10.1016/j.dib.2016.11.029 (PMC5126131; doi:10.1016/j.dib.2016.11.029)
Supplement: Supplementary file 1 — Supplementary material [file mmc1.docx]

**Conflict of Interest**

The authors have declared no conflict of interest.
